# Heart rate variability versus visual analog scale for objective and subjective mental fatigue detection: A randomized controlled trial

**DOI:** 10.1371/journal.pmen.0000240

**Published:** 2025-01-24

**Authors:** Hiroaki Yoshikawa, Yumi Adachi, Ayako Baba, Chiaki Takikawa, Yuya Yamaguchi, Wakana Nakai, Daiki Sudo

**Affiliations:** 1 Health Service Center, Kanazawa University, Kanazawa, Ishikawa, Japan; 2 Kanazawa Educational Yell Psychological Assistance Team (KEYPAT), Kanazawa University, Kanazawa, Ishikawa, Japan; 3 Communication Systems R&D Division, KYOCERA Corporation, Yokohama, Kanagawa, Japan; University of Turin, ITALY

## Abstract

Fatigue is a multidimensional phenomenon. Although psychological tests can be used to evaluate subjective fatigue, an objective measurement of fatigue is needed to evaluate mental, physical, and occupational health and inform appropriate interventions. Heart rate variability (HRV) has emerged as a potential candidate for assessing objective mental fatigue; however, its effectiveness and safety remain inconclusive. To address these, we conducted a single-center, randomized trial to compare the efficacy and safety of HRV with subjective psychological tests for evaluating mental fatigue. Participants aged 20 to 65 years who had received annual health check-ups and had been found to have no health concerns were included in this study. We compared HRV indexes after performing a calculation task with a control group that rested. The primary outcomes were HRV indexes as recorded by wearable electrocardiography after an intervention consisting of a calculation task. Subjective measures (visual analog scale [VAS] for fatigue and Profile of Mood States 2nd Edition [POMS2]) were evaluated as secondary outcomes. One hundred forty participants were randomized into a calculation task and control groups. Participants who performed the calculation task had a lower square root of the mean squared differences between successive RR intervals (RMSSD), absolute power in the high-frequency band, and standard deviation of Poincaré plot. In psychological tests, participants who performed the calculation task demonstrated significantly higher scores on the VAS for fatigue and POMS2. Multiple comparisons of RMSSD from HRV indexes, VAS for fatigue, and total mood disturbance from POMS2 revealed that RMSSD and VAS for fatigue were significant indicators associated with the calculation task. On the other hand, the vectors of HRV indexes and psychological tests differed based on primary component analysis. We identified RMSSD, an objective index, and VAS for fatigue, a subjective index, as significantly related to mental fatigue.

## Introduction

Fatigue is a phenomenon experienced in individuals even in healthy conditions [1–3]. Fatigue includes a feeling of tiredness, exhaustion, and lack of energy [4]. People experience fatigue in burnout [5], sports competition [6], overwork experienced by sustained shift work [7], or long-distance driving [8]. Fatigue is multidimensional, including mental, physical, social, and pathological factors, making it difficult to evaluate appropriately. In addition, people might not recognize their fatigue by themselves from subjective aspects [9].

One of the most critical factors in determining fatigue for research and clinical practices is its appropriate assessment. There are subjective and objective methods to evaluate the nature and severity of fatigue. Subjective methods include self-reporting questionnaires such as the visual analog scale (VAS) for fatigue [10, 11], the Fatigue Severity Scale [12], and the Multidimensional Fatigue Inventory [13]. However, mental fatigue includes difficulty concentrating, reduced alertness, impaired decision-making, and memory loss resulting from prolonged cognitive insufficiency [14]; therefore, the proper evaluation is complex from subjective dimensions. Objective methods for fatigue evaluation include heart rate variability (HRV) [15]; cognitive performance tests, such as evaluation of reaction time, attention span, working memory, and decision-making ability [16]; electroencephalography (EEG) [17, 18]; and functional magnetic resonance imaging (fMRI) [19]. These methods provide a more quantitative assessment but are more expensive. Besides, EEG or fMRI prevents participants from leading their daily lives during measurement. Therefore, HRV records using wearable devices are promising tools for monitoring fatigue.

HRV is the variation in the time interval between consecutive heartbeats, typically measured as the beat-to-beat changes in the RR intervals on an electrocardiogram (ECG). As utilization in health research, HRV assessments of mental fatigue during a cognitively demanding task were reported elsewhere [20]. It found that specific HRV parameters, particularly those reflecting vagal activity, were associated with increased mental fatigue. A review article provides valuable insights into using HRV as a stress measure in a demanding work environment focusing on the medical profession [21]. However, there has been a lack of randomized controlled trials (RCT) to detect mental fatigue using HRV. The solid evaluation using RCT of the HRV measurement for fatigue encourages the further utilization of HRV in fatigue research and clinical practices. In addition, there were limited comparative studies between subjective and objective fatigue indexes.

Therefore, we conducted an RCT to answer the efficacy and safety of HRV in measuring mental fatigue parallel to subjective indexes. We selected VAS for fatigue [10, 11] and Profile of Mood States 2nd Edition (POMS2) [10, 22] as subjective indexes. VAS is easy to understand and use, allowing for fine-grained distinctions in fatigue levels. It is also easily adapted to different contexts. Its simplicity, sensitivity, and flexibility make it a practical and informative option for research and clinical practices. While the VAS measures fatigue intensity, POMS2 helps understand the broader emotional context of fatigue. POMS2 acknowledges that fatigue is not just a physical sensation but can also be influenced by emotional and mental factors.

This study aimed at multidimensional analyses of fatigue from subjective and objective aspects using the RCT to provide a solid understanding of fatigue and expand future utilization of our combinatory evaluations in fatigue studies. We hypothesize that HRV could evaluate mental fatigue appropriately.

## Methods

### Study design

**Fig 1:**
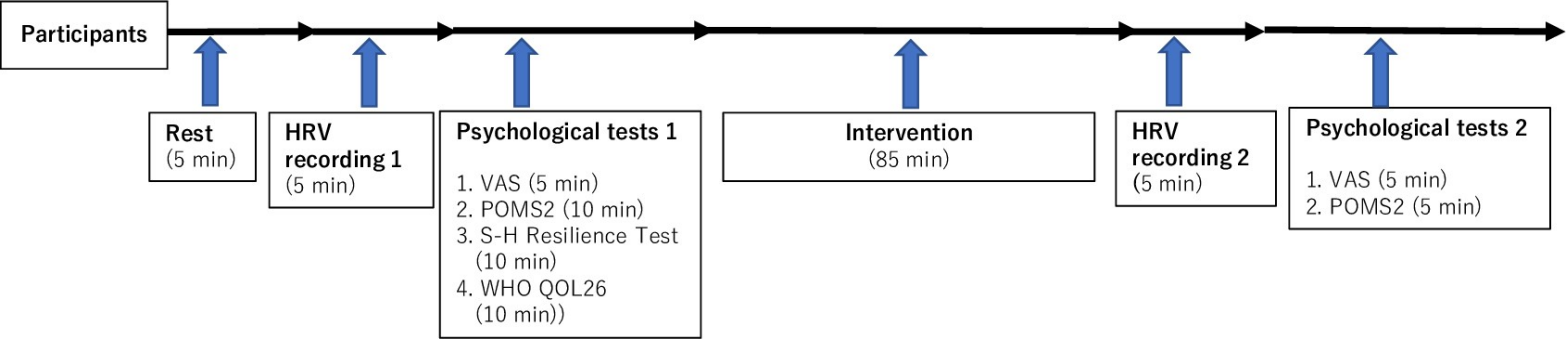
Trial design. Note. We tested participants according to this trial design. HRV: heart rate variability, VAS: visual analog scale, POMS2: Profile of Mood States 2nd Edition, S-H resilience Test: Sukemune-Hiew Resilience Test, WHO QOL26: World Health Organization Quality of Life 26.

This randomized study aimed to evaluate the efficacy of HRV in detecting mental fatigue (ISRCTN11112963). We recruited participants from the general population in Kanagawa, Japan. The study was conducted under the Good Clinical Practice guidelines and the Declaration of Helsinki. Fig 1 shows the overall assessment procedures.

### Standard protocol approvals, registrations, and participant consents

The Ethics Committee of Medicine, Kanazawa University, approved the study (2021–031). We obtained written informed consent from all participants enrolled before enrollment in the study. Participants received a comprehensive information sheet detailing the study’s purpose, procedures, potential risks and benefits, and their right to withdraw at any time. The research team was available to answer any questions. If participants reported feeling unwell during the study, we arranged for them to consult appropriate medical attention. All participant data was de-identified and stored securely, following data protection regulations.

### Participants

The inclusion criteria were: 1) receiving annual health check-ups and being found to have no health concerns, and 2) being aged 20–65 years. The exclusion criteria were: 1) having a cardiac pacemaker, 2) having arrhythmia, and 3) taking medicine that affects autonomic nervous functions such as a beta-blocker. We performed research procedures in quiet rooms with comfortable temperatures in the Kanagawa area. All procedures began around 9 a.m. and finished around noon. We prohibited the consumption of drinks containing caffeine or alcohol and smoking prior to the study. HRV indexes are susceptible to stress levels or physical activities. To remove these potential confounding factors, we asked participants to relax, and it took 45 minutes to explain the research and give informed consent. We also monitored the room temperature during the study and adjusted it to around 24°C.

### Interventions

#### Calculations

**Fig 2:**
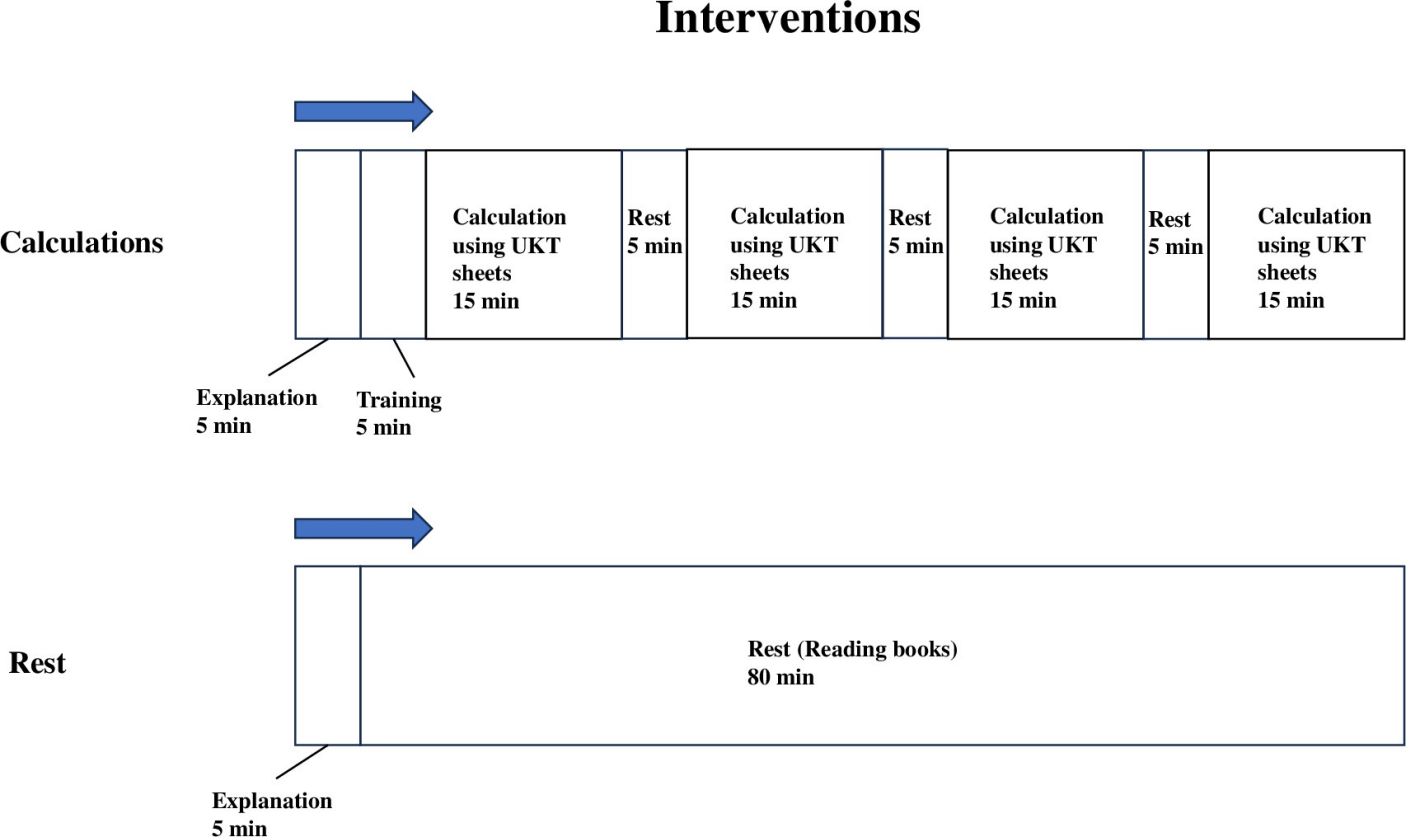
Experimental interventions. *Note*. The calculation task was administered using Uchida-Kraepelin test (UKT) sheets. Control participants were instructed to rest and provided with books to read with ease and calming content.

We used Uchida-Kraepelin test (UKT) sheets [23] for loading the calculation task. The UKT is a serial addition test in which participants are required to perform calculations as quickly and accurately as possible within 30 minutes. The test was conducted using a pre-printed paper containing 15 lines of random, single-digit, horizontally aligned numbers. Participants were instructed to start a new line every minute, regardless of their progress on the current line. Each line contained more calculations than could be completed within the minute, ensuring participants couldn’t finish before being prompted by the examiner to move to the next line. Typically, this test is performed in cycles of 15 minutes of work followed by 5 minutes of rest. For this study, four cycles were used (Fig 2). The UKT sheets were employed solely to induce mental fatigue; the scores were not evaluated.

#### Control

As a control, participants were instructed to take a rest and provided with easy and calming books to read. The rest period lasted 80 minutes, matching the calculation task’s duration (Fig 2).

### Outcomes

#### Primary outcomes

The primary outcome measures were HRV indexes. We used Polar H10 wearable ECG sensors and Polar Vantage V2 wristwatches (Polar Japan, Tokyo, Japan), enabling continuous RR interval recording. The Polar H10 wearable ECG sensor shows strong agreement and slight bias compared with conventional ECG recordings [24]. The recording time was five minutes (Fig 1). The collected RR interval data were transferred to a Windows personal computer and analyzed with Kubios (Kubios, Kuopio, Finland) [25]. We obtained time-domain, frequency-domain, and nonlinear indexes. We calculated the standard deviation of RR intervals (SDNN) and the square root of the mean squared differences between successive RR intervals (RMSSD) for the time-domain index. For frequency-domain indexes, we calculated power (ms^2^) of very low frequency (VLF) (0.00–0.04 Hz), low frequency (LF) (0.04–0.15 Hz), high frequency (HF) (0.15–0.40 Hz), and total power (sum of VLF, LF, and HF), as well as LF/HF ratio. We calculated standard deviations of the Poincaré plot (SD1, SD2), SD2/SD1 ratio, and short-term fluctuations of detrended fluctuation analysis (α1) for nonlinear indexes.

#### Secondary outcomes

To evaluate fatigue, we used VAS for fatigue, following the instructions of the Japanese Society of Fatigue Science. VAS for fatigue is a valid and reliable tool for measuring fatigue intensity, mainly when used alongside other fatigue assessments [26]. We also used Profile of Mood States 2^nd^ Edition (POMS2) Japanese version (Kaneco Shobo, Tokyo, Japan) [27]. POMS2 is a mood inventory containing 65 items that describe six different moods: Tension-Anxiety (TA), Depression-Dejection (DD), Anger-Hostility (AH), Vigor-Activity (VA), Fatigue-Inertia (FI), and Confusion-Bewilderment (CB). Total Mood Disturbance (TMD) score was calculated as ([AH + CB + DD + FI + TA]–VA). POMS2 offers insights into the emotional aspects of fatigue and their impact on mood, and it has been well-validated [28–30].

In addition, we used two psychological tests to assess resilience and quality of life (QOL). The Sukemune-Hiew (S-H) Resilience Test assesses the power of resilience, and it has been validated [31, 32]. We purchased printed test sheets from Takei Scientific Instruments Co. Ltd. (Niigata, Japan). A Japanese version of the World Health Organization QOL 26 (WHO-QOL26) was used to assess QOL [33, 34]. WHO-QOL26 measures QOL across four key domains (physical, psychological, social relationships, and environmental), and it has been validated [35].

### Sample size

Sample size was calculated using a two-sample t-test to detect a difference in the mean outcome between the counting task and control groups. We assumed a power of 80%, a significance level of 0.05, and a minimum detectable effect size of 18. The standard deviation of the outcome was estimated to be 40, based on a previous study [36]. We assumed a 1:1 ratio of intervention to control group members and a 10% dropout rate. The calculated sample size was 75 participants per group.

### Randomization

Participants were randomized in a 1:1 ratio to the intervention or control groups. We used a computer-generated random number sequence for simple random allocation. Allocation concealment was ensured using sequentially numbered, opaque, sealed envelopes prepared by an independent research team member.

### Statistical methods

Data was analyzed using JMP (Version 17.2, SAS Institute, Japan, Tokyo, Japan). Missing data was minimal (< 5%). Outliers were assessed using boxplots. Detected outliers were examined and retained if deemed genuine. The normality of distributions was evaluated using Shapiro-Wilk tests in conjunction with visual inspection of histograms. For normally distributed data, equality of variance was tested using a two-tailed F test. We used an independent two-sample, two-tailed t-test for the equal variance data, and Welch’s test for data with non-equal variance. For non-normally distributed data, we used Wilcoxon-Mann-Whitney test. Fisher’s exact test was conducted as a 2-tailed test. For multiple comparisons, we employed logistic regression analysis. We generated receiver operating characteristic (ROC) curves and calculated the area under the curve (AUC), as well as sensitivities, specificities, positive predictive value (PPV), and negative predictive value (NPV), to assess the significance of the indexes. Significance was defined as P < 0.05.

Principal component analysis (PCA) was applied to the dataset to reduce dimensionality and explore underlying patterns. Prior to PCA, the data were standardized. PCA was performed using JMP 17.2. The number of principal components to retain was determined by examining the scree plot and aiming for a cumulative explained variance of more than 80%. The selected principal components were rotated using the varimax method to enhance interpretability, and scores on the principal components were used for visualization.

## Results

### Participant flow

**Fig 3:**
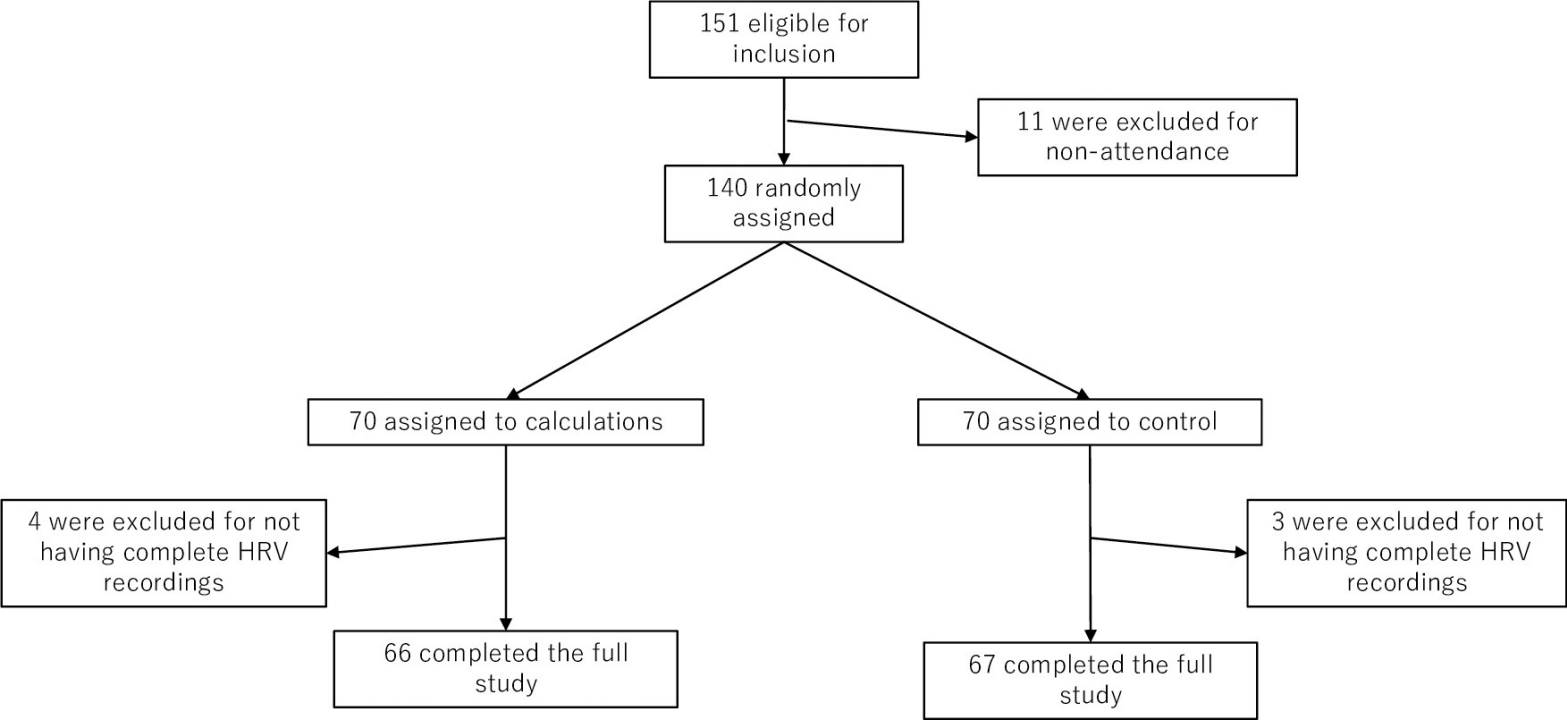
Participant flow diagram. *Note*. One hundred forty participants were randomized to calculation tasks or control groups. The studies were performed by per-protocol analysis.

One hundred forty participants were randomized (Fig 3). Participants were allocated randomly to the calculation task or control group. Four participants from the calculation task and three from the control group were excluded from the analyses because of incomplete recording of HRV. The studies were performed by per-protocol analysis.

### Recruitment

The first participant enrolled in June 2022, and the last participant finished the study in August 2022. Participants were followed up until September 30.

### Baseline data

Baseline data are shown in Table 1. POMS2 DD was significantly higher in the counting task group; however, there was no significant difference in TMD between the counting task and control groups.

**Table 1:** Baseline data.

	Calculation	Control	S	Z	P
**Number (men; women)**	66 (32:34)	67 (34:33)			0.8619^a^
**Age (year old)** median (IQR)	43 (29–53)	43.5 (31–54)	4551.5	0.588081	0.5614^b^
**History**	nyoma uteri (1) inguinal hernia (1)				
**Present illness**	None	acne (1)			
**HRV indexes** median (IQR)					
** SDNN**	22.9 (17.8–36.9)	27.6 (18.6–36.3)	4671	1.11829	0.2634 ^b^
** RMSSD**	18.2 (13.3–29.2)	22.3 (16.7–29.8)	4667	1.10029	0.2712 ^b^
** VLF (ms** ^**2**^ **)**	45.3 (20.9–81.5)	55.3 (26.7–103.1)	4667	1.10029	0.2712 ^b^
** LF (ms** ^**2**^ **)**	239.8 (136.2–664.8)	397.2 (141.6–853.1)	4645	1.00129	0.3167 ^b^
** HF (ms** ^**2**^ **)**	144.9 (71.0–295.4)	157.6 (74.3–352.2)	4493	0.31726	0.7510 ^b^
** LF/HF**	2.0 (0.9–4.0)	2.2 (0.8–6.4)	4603	0.81228	0.4167 ^b^
** Total power (ms** ^**2**^ **)**	503.0 (256.4–1087.1)	693.7 (302.7–1192.5)	4656	1.04179	0.2975 ^b^
** SD2**	30.2 (22.5–43.8)	33.2 (23.4–46.0)	4024	0.90678	0.3645 ^b^
** SD1**	12.9 (9.4–20.7)	15.8 (11.8–21.1)	4668	1.10479	0.2692 ^b^
** SD2/SD1**	2.2 (1.7–2.8)	2.3 (1.6–2.9)	4450	0.12375	0.9015 ^b^
** α1**	1.17 (1.00–1.47)	1.17 (0.94–1.46)	4504	0.36676	0.7138 ^b^
**VAS for fatigue** median (IQR)	3 (1.9–5.1)	3.1 (1.8–5.2)	4369	-0.23858	0.8114 ^b^
**POMS2** median (IQR)					
** Anger-Hostility (AH)**	40 (38–45)	39.5 (38–48)	4249	-0.79212	0.4283 ^b^
** Confusion-Bewilderment (CB)**	47 (42–53)	44 (40–50)	3092	-1.93573	0.0529 ^b^
** Depression-Dejection (DD)**	46 (42–50)	42 (40–48)	3902	-2.35881	0.0183 ^b^
** Fatigue-Inertia (FI)**	45 (39–50)	42 (37–49)	4198	-1.00937	0.3128 ^b^
** Tension-Anxiety (TA)**	46 (40–51)	43 (38–48)	3998	-1.91164	0.0559 ^b^
** Vigor-Activity (VA)**	51 (44–59)	55 (47–61)	4666	1.09495	0.2735 ^b^
** Friendliness (F)**	52 (45–59)	52 (47–59)	4446	0.10375	0.3174 ^b^
** Total Mood Disturbance (TMD)**	44 (40–49)	41 (39–49)	3995	-1.92290	0.0545 ^b^
**S-H resilience score** median (IQR)	102 (93–109)	106 (93–111)	4603	0.81046	0.4177 ^b^
**WHO QOL26** median (IQR)					
** Physical functioning**	3.71 (3.43–4.14)	3.86 (3.43–4.29)	4759	1.52005	0.1285 ^b^
** Psychological functioning**	3.33 (3–3.67)	3.5 (3–4)	4726	1.37056	0.1705 ^b^
** Social relationship**	3.33 (3–4)	3.33 (3–4)	4534	0.50959	0.6102 ^b^
** Environmental functioning**	3.63 (3.38–4)	3.75 (3.25–4.0)	4541	0.53273	0.5942 ^b^
** Global functioning**	3.5 (3–4)	3.5 (3–4)	4534	0.51075	0.6095 ^b^
** Average**	3.54 (3.27–3.85)	3.635 (3.27–4.05)	4659	1.06482	0.2820 ^b^

*Note*. IQR: interquartile range, SDNN: standard deviation of RR intervals, RMSSD: square root of the mean squared differences between successive RR intervals, VLF (ms^2^): absolute power for very low-frequency bands, LF (ms^2^): absolute power for low-frequency bands, HF (ms^2^): absolute power for high-frequency bands, Total power (ms^2^): absolute power for total power bands, SD1, SD2: standard deviations of Poincaré plot, α1: short-term fluctuations of detrended fluctuation analysis, S-H resilience score: Sukemune-Hiew resilience score, WHO: World Health Organization, QOL: quality of life, S: Sum of ranks (Rank sum), Z: Standardized test statistics (Z-score)

^a^Fisher’s exact test (2-tail)

^b^Wilcoxon-Mann-Whitney test

### Numbers analysed

There were 66 participants in the calculation task group and 67 in the control group.

### Outcomes and estimation

#### Primary outcomes

RMSSD, HF, and SD1 were significantly lower and SD2/SD1 were significantly higher in calculation task group (Table 2).

**Table 2:** Primary outcomes (HRV indexes).

HRV indexes	Calculation task median (IQR)	Control median (IQR)	S	Z	P
**SDNN**	29.2 (23.2–37.2)	33.4 (23.1–45.7)	4802	1.70781	0.0877
**RMSSD**	24.3 (16.0–31.4)	28.6 (20.9–42.5)	4957	2.40534	0.0162
**VLF (ms** ^**2**^ **)**	65.0 (34.1–100.6)	71.4 (28.5–106.3)	4650	00.3059	0.3059
**LF (ms** ^**2**^ **)**	430.2 (206.5–938.3)	435.7 (250.2–1127.4)	4547	0.56027	0.5753
**HF (ms** ^**2**^ **)**	180.0 (94.1–314.8)	255.7 (145.1–621.0)	4888	2.09483	0.0362
**LF/HF**	2.6 (1.4–4.8)	1.8 (0.8–4.0)	4081	-1.53231	0.1254
**Total power (ms** ^**2**^ **)**	676.8 (481.8–1410.8)	987.4 (472.5–1918.6)	4759	1.51437	0.1299
**SD2**	35.9 (29.2–47.8)	41.3 (29.3–60.3)	4707	1.28030	0.2004
**SD1**	17.2 (11.3–22.3)	20.2 (14.8–30.1)	4957	2.40534	0.0162
**SD2/SD1**	2.3 (1.7–3.0)	2.0 (1.4–2.8)	3982	-1.97782	0.0479
**α1**	1.2 (1.0–1.5)	1.2 (0.9–1.3)	4056	-1.664481	0.1000

*Note*. IQR: interquartile range, SDNN: standard deviation of RR intervals, RMSSD: square root of the mean squared differences between successive RR intervals, VLF (ms^2^): absolute power for very low-frequency bands, LF (ms^2^): absolute power for low-frequency bands, HF (ms^2^): absolute power for high-frequency bands, Total power (ms^2^): absolute power for total power bands, SD1, SD2: standard deviations of Poincaré plot, α1: short-term fluctuations of detrended fluctuation analysis, S: Sum of ranks (Rank sum), Z: Standardized test statistics (Z-score)

Wilcoxon-Mann-Whitney test

#### Secondary outcomes

VAS for fatigue and POMS2 (CB, DD, FI, TA, TMD) were significantly higher in calculation task group than the control group (Table 3).

**Table 3:** Secondary outcomes (Psychological tests).

	Calculation task median (IQR)	Control median (IQR)	S	Z	P
**VAS for fatigue**	5.2 (4.1–6.9)	3.7 (2.8–5.7)	3597	-3.71106	0.0002
**POMS2**					
** Anger-Hostility (AH)**	39 (38–43)	38 (38–40)	4058.5	-1.77659	0.0757
** Confusion-Bewilderment (CB)**	47 (42–53)	42 (38–47)	3782	-2.88211	0.0040
** Depression-Dejection (DD)**	43 (40–49)	41 (40–44)	3861	-2.60445	0.0092
** Fatigue-Inertia (FI)**	46 (41–54)	41 (37–50)	37315	-3.11499	0.0018
** Tension-Anxiety (TA)**	45 (41–49)	40 (36–46)	3633	-3.555533	0.0004
** Vigor-Activity (VA)**	48 (40–56)	51 (44–59)	4632.5	0.94594	0.3442
** Friendliness (F)**	47 (40–59)	50 (39–59)	4607.5	0.83377	0.4044
** Total Mood Disturbance (TMD)**	45 (41–49)	40 (38–45)	3722.5	-3.15001	0.0016

*Note*. IQR: interquartile range, VAS: visual analog scale, POMS2: Profile of Mood States 2nd Edition, S: Sum of ranks (Rank sum), Z: Standardized test statistics (Z-score)

Wilcoxon-Mann-Whitney test

### Multiple comparisons of indexes

HRV indexes were derived from the same RR interval data, introducing potential confounding factors. Additionally, subitems from POMS2 are related to TMD, presenting further confounding factors. We performed multiple comparisons between the Calculation task and Control groups, using RMSSD from HRV indexes, VAS for fatigue, and TMD from POMS2 to account for this. RMSSD and VAS for fatigue differed significantly between the Calculation task and Control groups (Table 4).

**Table 4:** Multiple comparisons of RMSSD, VAS for fatigue, and POMS2 (TMD).

	odds ratio	Chi-square	95% CI	P
**RMSSD**	1.03	6.80	1.007–1.055	0.0091
**VAS for fatigue**	0.73	9.66	0.582–0.890	0.0019
**POMS2 Total Mood Disturbance** **(TMD)**	0.96	2.42	0.913–1.010	0.1197

*Note*. VAS: visual analog scale, POMS2: Profile of Mood States 2nd Edition, CI: confidence interval

Nominal logistic regression analysis, Whole Model Test: ChiSquare; 23.61484, P < 0.0001

### Specificity, sensitivity, PPV, and NPV of RMSSD and VAS for fatigue in predicting mental fatigue

**Fig 4:**
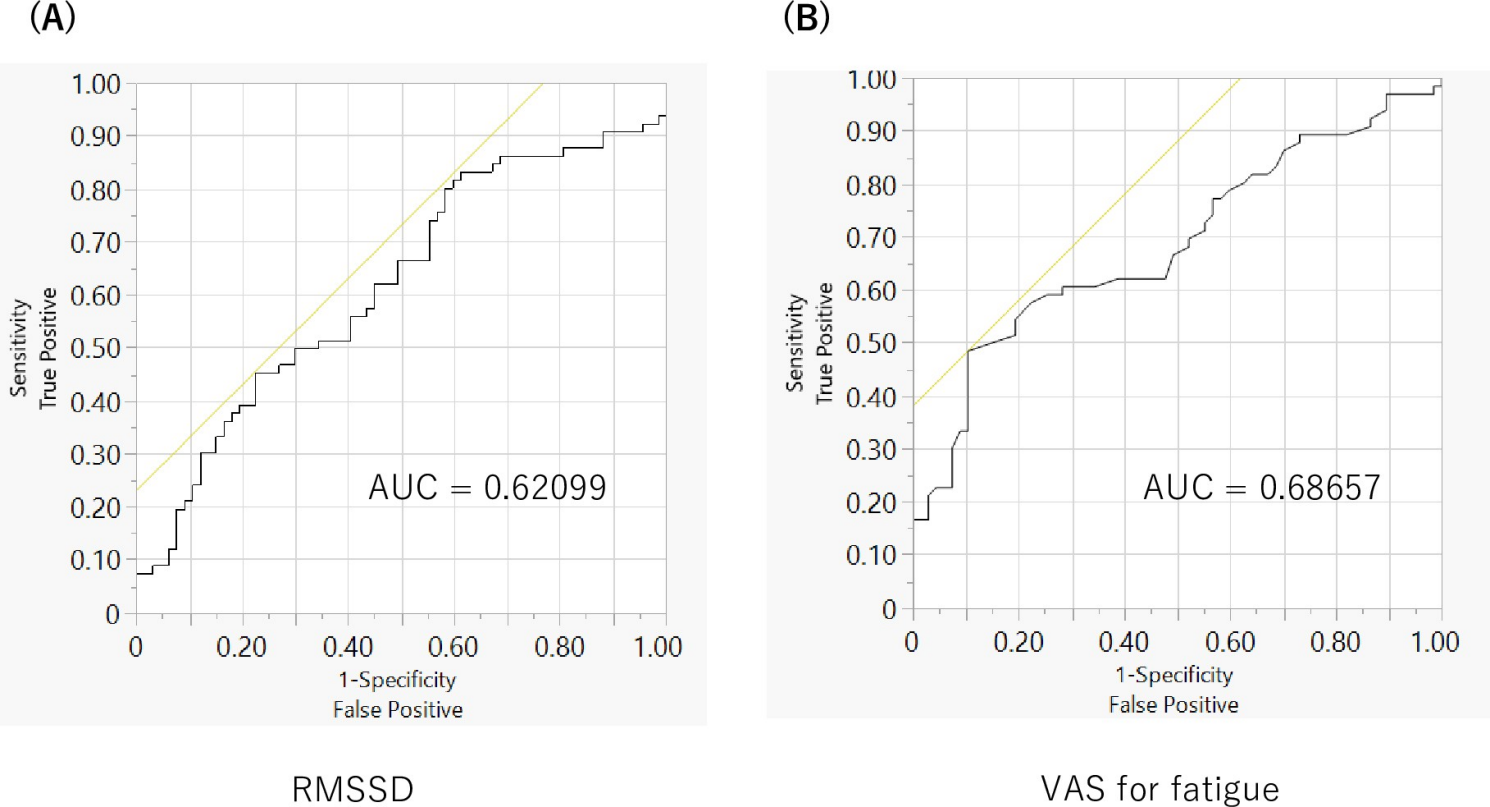
ROC curves of RMSSD and VAS for fatigue. *Note*. A: ROC Curve from RMSSD. The AUC was estimated as 0.62099. B: ROC Curve from VAS for fatigue. AUC was estimated as 0.68657.

We drew ROC curves and estimated AUC, sensitivity, and specificity from RMSSD and VAS for fatigue data. The AUC values from RMSSD and VAS for fatigue were similar (Fig 4). The cut-off value for RMSSD was 31.69530, and that for VAS for fatigue was 3.5.

Specificity, sensitivity, PPV, and NPV were similar between RMSSD and VAS for fatigue (Table 5).

**Table 5:** Specificity, sensitivity, PPV, and NPV of RMSSD and VAS for fatigue.

	Specificity	Sensitivity	PPV	NPV
**RMSSD**	0.7761	0.4545	58.62%	66.67%
**VAS for fatigue**	0.8955	0.4848	63.16%	83.78%

*Note*. RMSSD: square root of the mean squared differences between successive RR intervals, VAS: visual analog scale

PPV: positive predictive value, NPV: negative predictive value

### Ancillary analyses

#### PCA of HRV, VAS for fatigue, and POMS2

**Fig 5:**
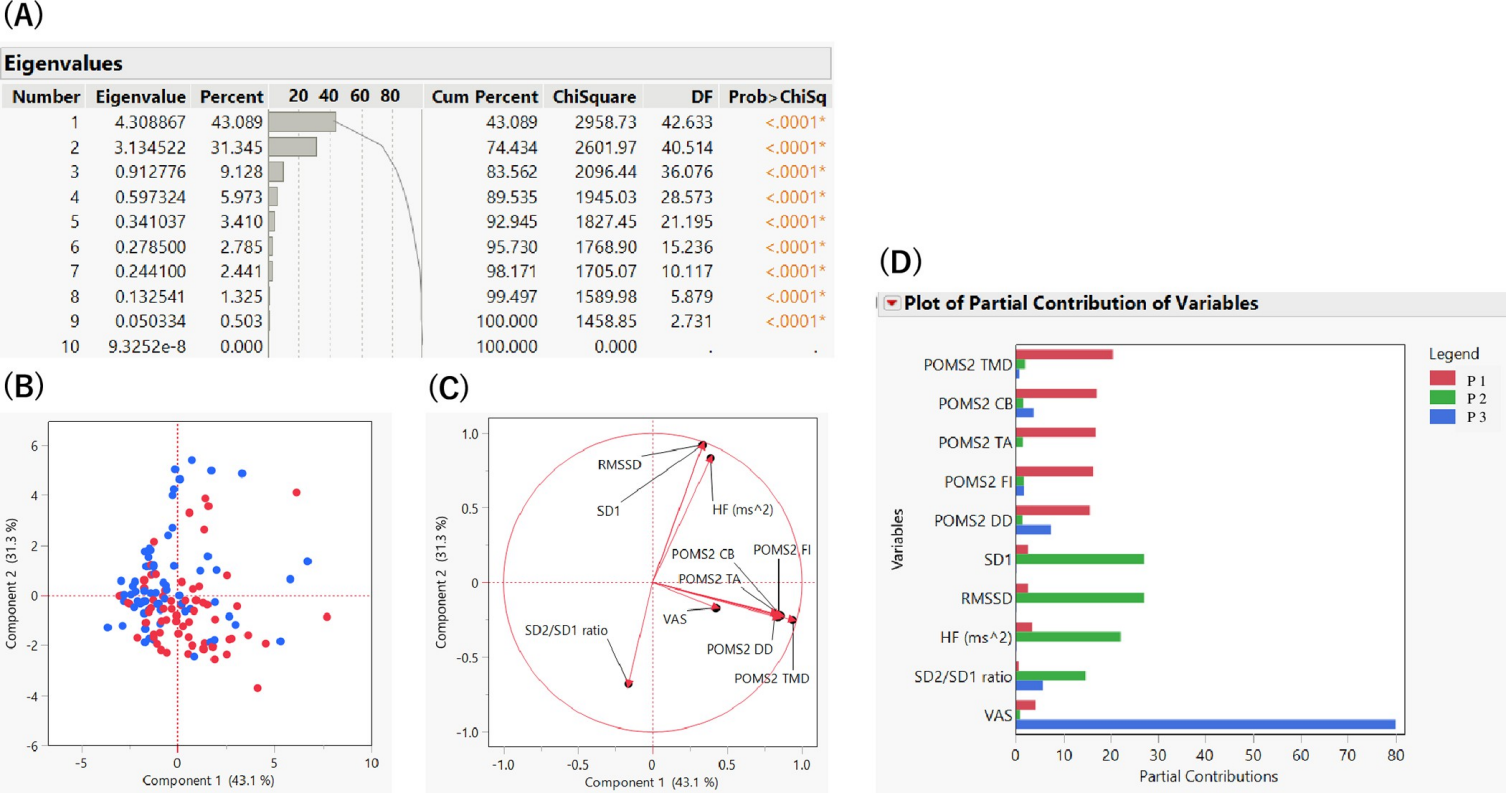
Principal component analyses of HRV, VAS for fatigue, and POMS2. *Note*. A: Eigenvalues and percentages of variances. B: Score plot of Components 1 and 2. Red dots indicate the calculation task, and blue dots indicate control. C: Loading plot of components 1 and 2. D: Plot of the partial contribution of variables. POMS2: Profile of Mood States 2nd Edition, TMD: Total Mood Disturbance, CB: Confusion-Bewilderment, TA: Tension-Anxiety, FI: Fatigue-Inertia, DD: Depression-Dejection, SD1: Standard deviation 1, SD2/SD1: ratio of Standard deviation one and Standard deviation 2, RMSSD: square root of the mean squared differences between successive RR intervals, HF (ms^2^): high-frequency power, VAS: visual analog scale, P 1: Principal component 1, P 2: Principal component 2, P 3: Principal component 3.

We conducted PCA to explore patterns within the dataset, focusing on significant factors after the intervention. PCA revealed that the first three components had higher eigenvalues and accounted for 83.562% of the total variance; thus, we proceeded with these three components for further analysis (Fig 5A). The score plot using components 1 and 2 showed that the distribution of the calculation task (red dots) and rest (blue dots) was similar (Fig 5B). The loading plot indicated that POMS2 and VAS for fatigue had similar vectors, whereas HRV indexes had distinct vectors (Fig 5C). The partial contribution plot revealed that principal component 1 primarily represented POMS2 indexes, principal component 2 represented HRV indexes, and principal component 3 represented VAS for fatigue (Fig 5D). Despite being psychological tests, POMS2 and VAS for fatigue exhibited different specificities.

#### Harms

Participants were not exposed to subjective or objective harm during and after the trials.

## Discussion

HRV has been extensively studied to evaluate the autonomic nervous system function [37]. It also has been used to assess cardiovascular health [38], mental health [39], and diseases, including chronic fatigue syndrome and posttraumatic stress disorder [40].

HRV has at least three analysis methods: 1) Time Domain Indexes such as SDNN and RMSSD. RMSSD is strongly influenced by parasympathetic activity; 2) Frequency Domain Indexes: VLF, LF, and HF power (ms2). HF represents parasympathetic activity, and LF reflects the sum of sympathetic and parasympathetic activities; 3) Non-Linear Indexes: SD1, SD2, and α1. SD1 is similar to HRV metrics with RMSSD [41]. As no single index precisely represents HRV, we need to select an appropriate index that fits the purpose of the study. LF/HF from frequency domain indexes represents sympathetic activity. In a previous study, we found that university students with mental distress had a higher LF/HF ratio even at rest, indicating that their sympathetic nervous tone remained elevated [32].

HRV has unique characteristics: 1) HRV baselines vary considerably from person to person. This variation makes it more beneficial to follow trends in individuals; 2) HRV is susceptible to external factors, including age, sex, caffeine, alcohol, and medication intake; and 3) HRV values can fluctuate significantly due to circadian rhythms and other physiological processes.

Our study tested VAS for fatigue and POMS2 simultaneously and found that these psychological tests evaluated fatigue from different perspectives than HRV. A wearable chest ECG sensor is ideal for collecting HRV data. Advances in sensor technology and artificial intelligence have enhanced the value and reliability of HRV measurements. Future studies should focus on developing methods to evaluate personal variations in fatigue levels.

### Limitations

Our study was conducted in a single center, and the number of participants was relatively small. In future studies, we should consider the more diverse participants across multiple centers. Measurement of HRV using our chest strap-type simple ECG may cause discomfort to the participants. In the future, we need to develop a method to measure ECG for a long time without restraint. We set specific criteria for recruiting research participants, such as having them undergo annual health check-ups and being found to have no health concerns. It is necessary to consider whether these criteria may have influenced the study results. The VAS for fatigue and POMS2 are self-reported measures. Therefore, there might be potential constraints.

### Generalizability

Our methods of hybrid evaluation of fatigue from subjective (VAS for fatigue) and objective (HRV) aspects are promising in future research and clinical practices. HRV indexes are representative of the autonomic nervous system that supports our lives. HRV analysis using wearable devices is the ideal tool to monitor the autonomic function of humans longitudinaly and non-bindingly, which makes it possible to monitor interventional-based HRV monitoring. In addition, the appropriate combination of subjective indexes makes our methods worthwhile in various situations.

Excessive fatigue increases the risk of accidents [42]. Cultural differences in the perception and reporting of effort and pain prevent workers from accurately estimating fatigue [43]. Workers become stressed, sad, isolated, and unaware of this; they place themselves at risk for burnout and their customers at risk for suboptimal services [9]. In addition, environmental changes brought by disasters also prevent people from accurately recognizing fatigue. On January 1, 2024, a moment magnitude (Mw) 7.5 earthquake struck the Noto Peninsula of Ishikawa Prefecture, Japan (the 2024 Noto Peninsula Earthquake) [44]. The epicenter was 120 km from our institution. Due to the peninsula’s geography, roads were cut off, and the ports were rendered unusable, delaying reconstruction. A year later, the restoration is still underway. We formed the Kanazawa Educational Yell Psychological Assistance Team (KEYPAT), which supports the mental health of earthquake victims, on January 5, 2024 (X account: @KEYPAT468342). We often visited severely damaged areas and collaborated with the local supporters. Many earthquake victims were unaware of their emotional injury; therefore, objective evaluations were necessary. In these conditions, we can use HRV monitoring and psychological testing to find victims who need urgent mental intervention even if they do not realize their fatigue.

### Interpretation

In the present study, PCA revealed that objective and subjective fatigue have distinct vectors, indicating that they are independent markers. HRV control is closely related to functional brain connectivity. Studies have reported that higher HRV is associated with more robust functional connectivity with the default mode, salience, and central autonomic networks [45–47].

Regarding specific brain regions, HRV is linked to functional connectivity in areas of the anterior cingulate cortex, insula, amygdala, and prefrontal cortex, which have roles in emotion, attention, decision-making, and the autonomic nervous system [48, 49]. These frameworks suggest a bidirectional connection between brain regions that control heart rate via the autonomic nervous system and brain regions involved in processing sensory, emotional, and cognitive information. Further studies are necessary to understand the mutual interaction between heart rate control and brain functional connectivities.

## Supporting information

S1 Checklist CONSORT checklist. (DOCX)

S1 Data Minimal data for the study. (XLSX)

S1 Text Research protocol original. (PDF)

S2 Text Research protocol English. (PDF)
