# Supplementary material for: Heart rate variability versus visual analog scale for objective and subjective mental fatigue detection: A randomized controlled trial
Source: PLOS Ment Health. 2025 Jan 24;2(1):e0000240. doi: 10.1371/journal.pmen.0000240 (PMC12798607; doi:10.1371/journal.pmen.0000240)
Supplement: S1 Text — (PDF) [file pmen.0000240.s003.pdf]

2022.02.22 変更申請 ver.2.2

研究計画書

１. 課題名：心拍変動解析による心身の状態把握に関する研究

２. 研究の概要・目的・意義

心身の健康を維持しつつ修学や就労にあたることは、ストレスの多い現代社会において重要な課題となっている。特に新型コロナウイルス流行により、修学・就労の形態が大きく変わり、それに関連したメンタルヘルスの維持や改善が課題になっている。ところで我々の大学生を対象にした研究によって、メンタルヘルスに不調をきたす学生には、次の特徴があることを明らかになった。1)正常範囲ではあるが，ワーキングメモリが低い傾向がある，2）特に男性において，自閉スペクトラム障害の特性が高い，3）レジリエンスが弱い，4）性格的に不安特性が高い，5）男女ともに，社会生活におけるクオリティーオブライフ（QOL）を感じにくい，6）男性においては，メンタルヘルスにおける QOL の指標が低い，7）自律神経機能における交感神経の緊張が安静時でも高い [1]。この結果を踏まえて、今回の研究計画は、1)労働者のメンタルヘルスのモニタに有効な心拍変動の指標を明らかにすること、2)ミリ波センサによる非拘束型の心拍記録装置（共同研究者：京セラ株式会社開発）が従来の心臓の電気活動を解析する装置と同等の性能を有するか検討することを目的とする。なお、この装置は、将来的に市販化される場合、「研究用機器」として扱われる。またこの研究は、薬機法に基づく医療機器の性能評価を目的とするものではない。

３. 研究の科学的合理性と根拠

本研究は、侵襲性のない介入を用いたランダム化比較研究である。介入として精神的労作負荷（計算）を用いる。メンタルヘルスに不調をきたした学生の特性に関しては、先に述べたとおりであるが [1]、その中でもストレス下における業務遂行に関わる能力として重要なものはレジリエンスである。また業務を行う上で疲労と活力も重要な指標と考え、これらの項目を心理検査により評価する。

心拍変動解析を、今回の研究の評価に用いる根拠としては、次の報告に基づく。Porges, S.W.の提唱する Polyvagal theory によると、新しい有髄神経から構成される迷走神経の活動は、人の安心と社会的関りを豊かにし、結果としてレジリエンスを向上させる [2]。これらの知見から、我々は特に副交感神経活動に注目して、心拍変動解析を実施する計画を立てた。

心拍変動解析の方法としては、以前から研究され学会ガイドラインもある心電図波形の R-R 間隔の解析による自律神経機能解析がある [3]。しかし、心電計を装着したまま日常生活を送ることは困難であるため測定場所や条件が限定されていた。そこでミリ波センサを用いた装着の必要がなり心拍変動記録装置（共同研究者：京セラ株式会社が開発）を用いて、従来型の心電図記録装置と同様の測定結果が得られるかも検討する。

4. 研究対象者及び選定方針

（1）　適格基準

① 人材派遣会社に依頼して派遣された毎年健康診断を受け、健康上大きな問題のない者

② 本研究の参加に関して同意が文章で得られる者

③ 同意取得時の年齢が 20 歳以上の者

④ 男女同等の比率になるよう組入れる（研究対象者を派遣する人材派遣会社に、男女同数になるよう依頼）

（2）　除外基準

① 心臓ペースメーカを装着している者

② 不整脈を疾病として持つ者

③ 自律神経機能に影響を与える薬剤（βブロッカー）を服用している者

5. 目標数と研究実施期間

（1）目標数 140 例 ランダムに介入（連続加算作業）群とコントロール（読書）群に割り付ける。

（2）研究期間

研究期間:　承認日～2025 年 3 月 31 日

登録期間:　承認日～2022 年 9 月 30 日

観察期間（追跡期間）:　登録終了後 1 年間

解析期間:　承認日～2025 年 3 月 31 日

＊共同研究機関は、各機関の規定に従って倫理審査委員会の審査を受け、研究機関の長の許可を得る。

共同研究機関の研究開始日は，各機関における研究実施許可日とする。

6. 研究方法

（1）研究のデザイン

単一施設による前向きランダム化比較研究

（2）研究の方法

研究遂行にあたっては、金沢大学保健管理センター（以下、金沢センター）に研究本部を置き、データの管理、解析を行う。被験者の募集は、京セラ株式会社みなとみらいリサーチセンター（横浜市、以下、横浜センター）において、人材派遣会社に委託する。データの取得は、

横浜センターの建物内もしくはレンタルオフィスの室温が一定の部屋で実施する。金沢センターの研究代表者、研究分担者は、必要に応じてテレビ会議により、研究遂行の指導等を行う。横浜センターでは被験者の個人情報と取得データを管理し、管理台帳（電子データ）は鍵のかかる保管戸棚にノート PC もしくは記憶メディアに入れて保管する。共同研究機関である京セラの研究責任者または研究分担者が、説明と同意の取得を文書により行い、不明なことがあればテレビ会議で金沢センターの研究代表者もしくは研究分担者が回答する。介入（85 min）における 1. 連続加算作業と 2. 対照課題（読書）の選択は、くじ引きにより行う。なお、対照課題の読書は、負担の少ない小説（例：宮沢賢治、童話集）とし、金沢センターで選定して、横浜センターに郵送する。研究過程において、同一の書籍を使用する。

金沢センターでは、心理テストの解析と評価、心拍変動データの解析と評価を行う。解析結果は、横浜センターとファイルサーバを使い共有する。ファイルサーバはユーザーごとに、ユーザー名、バスワードを設定し、アクセスログを記録する。なお、ファイルサーバには、個人を特定できる情報は保存しない。

心拍変動解析に用いる測定器は以下の2機種を用い、同時記録する。

1） Polar 社　POLAR H10 心拍センサー（資料 1）と POLAR VANTAGE V2 スポーツウオッチ（資料 2）.
    （ア） 胸部センサによる簡易心電計であり、心電図の R-R 間隔を記録する。そのデータが心電計と同等の精度を持つことは検討済みである [4, 5]。
2） 京セラ株式会社　ミリ波センサ
    （ア） ミリ波レーダー技術を用いた非接触型の心拍記録計である（資料 3）。（なお、本装置は、開発中のもので高度の機密技術を含むため、仕様は提供できない）。

研究対象者に対する測定計画は、資料 4 を参照。

研究の進行に伴い、研究方法が追加されることがある。

研究対象者への心理テスト結果の開示は行わない。その理由としては、1） 心理検査の結果は，心理師による直接の説明でなければ適切に伝わらない、2） 限られた目的の心理検査結果を伝えることで誤った自己理解につながる，といった懸念があるためである。

7．観察・検査・報告項目

1） 研究対象者の特性に関する情報

年齢、性別、身長、体重、既往歴（慢性頭痛の有無等）、治療中の疾病、服用薬

2） 心理テスト

研究の観察、検査スケジュール（資料 4）を参照

3） 心拍変動

胸部装着型センサ（POLAR H10）ならびにミリ波センサより取得。

測定の際には、横浜センターの研究責任者または研究分担者が同席し、計画に従って

研究が進むよう工程管理をする。実施の前には、金沢センターと横浜センターで、十分な打ち合わせをして、手順に間違いのないようにする。記入した検査用紙は対応表を作成し、匿名化後、金沢センターへ郵送する。研究分担者の足立、馬場は質問紙の解析、点数化を行う。研究代表者の吉川は、心拍変動のデータを解析ソフトウェア kubios と京セラ株式会社の開発する解析アルゴリズムを使用して解析する。なお、測定データはファイルサーバを介して、京セラセンターと共有する。なお、金沢センターの研究責任者および研究分担者が、研究が適切に行われていることを確認するため、横浜センターにおいては研究実施の際に動画を撮影し、金沢センターと共有する。

8. 有害事象の評価と報告

（1）有害事象の定義と報告方法

□該当なし

■該当あり

本研究は侵襲のない軽度の介入（連続加算作業）を伴う前向き研究であるが、本研究による介入によって、有害事象が生じた際は必要な措置を行うとともに、部局長に報告する。

（2）研究対象者に生じる負担並びに予測されるリスク及び利益、これらの総合的評価並びに当該負担及びリスクを最小化する対策

①予測される利益

本研究に参加することによる研究対象者個人への直接的な利益はない。研究の成果は労働者の心身の健康モニタリングツールの開発に有益となる可能性がある。

②予測されるリスクと不利益

本研究は、侵襲のない軽度の介入（計算作業）によるランダム化比較試験であり、リスクは少ないと考えている。不利益としては、一人当たりの測定が約 200 分の時間を要するため、貴重な時間が失われることになる。実験の途中で水以外の飲食は禁止する。必要においてトイレに行くことは、測定や介入の合間に可能である。研究は室温や照度が一定で雑音やストレスがない環境の室内で行われるため、参加することによる健康リスクは少ないと考えられる。研究実施場所までの交通費が発生する場合は、研究対象者の自己負担とする。

万が一、研究実施中に研究対象者に健康被害が発生した際は試験を中断し、別室で休ませる。改善しない場合は近隣の医療機関を利用し、費用が発生する場合は、京セラ株式会社が負担する。

9. 評価項目

（1）主要評価項目：

心拍変動（Heart Rate Variability, HRV）解析結果

（2）副次評価項目：
1） POMS2 の点数
2） VAS 法による疲労度評価（資料 5）
3） S-H 式レジリエンス検査の点数
4） WHO QOL26 の点数

検定統計量の定義（添付の測定計画補足資料を参照）
・心理検査得点：心理検査-1、心理検査-2 のスコア、並びにその差分である。
・心拍変動解析の指標：心拍変動データは試験開始（観察期間（安静座位：5 min））から試験終了まで連続して計測する。検定統計量としては、記録 1、記録 2 それぞれの区間におけるパワースペクトラム解析値、およびその差分である。

１０. 統計的事項
本研究における目標数の設定は、我々の過去の研究結果[1]から実験計画統計により求めた。この研究では、対応のない２群間の比較を行った。データが正規分布をしているか否か調べたのち、等分散性か否かを確認した。正規性分布かつ等分散性の場合は、Student t-testを行い、等分散性ではない場合は Welch’s t-test を行った。非正規分布をするデータについては、Wilcoxon-Mann-Whitney test を実施した。
解析項目は、1） 心拍変動解析の指標ならびに心理検査得点の介入の前後による変化、2） 心拍変動解析の指標の Polar H10 とミリ波センサの相動性の評価である。1)における統計的方法は、対応のある２群間の比較を行い、統計的方法については、データの正規性を確認の後、パラメトリックもしくは非パラメトリックに対応した適正な統計解析方法を選択する。2)に関しては相関分析を実施する。

１１. 症例報告書の記入と報告
既定のものはない。研究工程と時間管理をするための、チェック表を作成する。

１２.倫理的配慮
（1）遵守する倫理指針や法令
本研究に携わるすべての者は、人を対象とする全ての医学研究が準拠すべき「世界医師会ヘルシンキ宣言」及び「人を対象とする医学系研究に関する倫理指針」（文部科学省・厚生労働省）の内容を熟読し理解した上で遵守し、研究を施行する。

（2）個人情報の保護の方法
研究に関わる関係者は、研究対象者の個人情報保護について、適用される法令、条例を遵守する。 また関係者は、研究対象者の個人情報およびプライバシー保護に最大限の努力を払い、本研究を行う上で知り得た個人情報を正当な理由なく漏らしてはいけない。

関係者がその職を退いた後も同様とする。

研究実施に係る試料等を取り扱う際は、被験者の秘密保護に十分配慮する。横浜センターから心理テスト（紙や冊子体）を金沢センターに送付する場合は、番号を使用し、被験者の個人情報が外部に漏れないよう十分配慮する。個人情報および匿名化した対応表は、それぞれ別にノート PC に電子ファイルとして保管し、そのノート PC は施錠された横浜センターの机に保管し，漏洩・盗難・紛失等が起こらないように厳重に管理する。個人情報が含まれない測定データは、ファイルサーバ上に保管し、それにアクセスするユーザーごとにパスワードを設定するとともにアクセスログを管理する。個人情報管理者として、馬場絢子（金沢大学保健管理センター）を置く。

学会などで研究結果を公表する際には個人が特定できないように配慮し，匿名性を守る。

研究の中止又は終了後，学会発表，論文発表のうち，最も遅い時期から，研究に関する電子データ及び実験・観察ノートは 10 年，その他研究データ等は5年保存する。

13．インフォームド・コンセントを受けるための手続きについて

京セラ株式会社の研究責任者または研究分担者は、事前に倫理審査委員会で承認の得られた同意説明文書を研究対象者に渡し、文書による十分な説明を行い、研究に参加するかどうかについて、研究対象者の自由意思による研究参加の同意を文書で得る。説明が不足する場合は、テレビ会議により、本学研究責任者又は研究分担者が説明を補う。

14．研究対象者に生じる費用負担について

研究対象者には経済的負担は生じないと考えられる。規定に従い謝礼を支払う。

15．本研究に係る資金ならびに利益相反について

本研究は京セラ株式会社との共同研究であり、費用は同社が負担する。研究に使用する機器（京セラ社製、ミリ波センサ）は、京セラ株式会社から提供される。本研究の実施および発表に際しては、利益相反関係を適切に管理し、中立性と公明性を維持して適正に研究を維持させる。また、本研究の研究担当者は、「金沢大学臨床研究利益相反マネージメントポリシー」に従い、金沢大学臨床研究利益相反マネージメント委員会に必要事項を申告し、その審査と承認を得るものとする。研究結果の公表に際しては、結果を発表する学会および雑誌の指針を遵守し、自己申告によって正確な状況を開示するものとする。

16．実施計画の変更について

研究の進捗にともない、研究内容及び研究組織・期間などに計画の変更の必要が生じた場合は、医学倫理審査委員会の承認を得て、変更を行う。

17．試料・情報について

（1）試料・情報の種類、保存、記録、破棄について

A. 人体から取得した試料

■該当なし

☐該当あり

試料の種類：

保存・破棄について：

試料及び情報の二次利用について：

保存の責任者について：

B. 情報

☐該当なし

■該当あり

情報の種類：年齢、性別、身長、体重、既往歴、服用薬などの研究対象者の基本データ（電子データ）、心理テスト調査票（紙データ、電子データ）、心拍変動解析測定データ（電子データ）

横浜センターでは、氏名、電話番号を取得する。

保存・破棄について：

研究責任者は、定められた保管方法に従って研究分担者等が適切に保管するよう指導し、情報の漏えい、盗難、紛失等が起こらないよう必要な管理を行う。電子データ及び実験・観察ノートは研究終了若しくは中断または，論文等が発表されてから遅い時期から 10 年間，その他の研究データ等は 5 年間保存した後，破棄する。情報の提供元の共同研究機関（横浜センター）においては、そのルールに基づき、情報を適切に保存及び破棄する。

試料及び情報の二次利用について：

本研究で得られた研究対象者の試料・情報は、同意を受ける時点では特定されない将来の研究のために用いる可能性がある。その場合には、新たな研究計画について本学倫理審査委員会の審査を受けたうえで、別途研究対象者に説明した上で実施する。

保存の責任者について：情報は研究責任者　吉川弘明　が保管する。

（2）試料・情報の他機関との授受の記録について

【他機関に試料・情報を提供する場合（業務の一部委託による提供を含む）】

■該当なし

□該当あり

①提供記録の作成方法

＊提供については、必ず提供先と相談の上、チェックすること。

□1）本研究計画書を提供記録とし、変更時は変更申請で対応する。

（上記の場合は、必ず説明文書に提供目的等を記載のこと）

□2）任意様式※を提供記録とし、「その他報告」か「実施状況報告」で対応する。

＊この際は報告する様式を添付すること

※厚労省 HP の様式を参考にすること。

http://www.mhlw.go.jp/stf/seisakunitsuite/bunya/hokabunya/kenkyujigyou/i-kenkyu/

□3）新規申請時は、本研究計画書を提供記録とするが、その後は 2）の対応とする。

＊この際は報告する様式を添付してください

□4）その他（具体的に：例：「提供に関する契約書（MTA（material transfer agreement）、DTA（data transfer agreement）等）」を用いる。 ）

②提供記録の保管方法

・提供記録の保管場所：

① 提供先の機関名称：

② 提供先の責任者名：

③ 提供する試料・情報の項目：

【他機関から試料・情報の提供を受ける場合】

□該当なし

■該当あり

①提供記録の作成方法

＊提供については、必ず提供元と相談の上、チェックしてください

■1）本研究計画書を提供記録とし、変更時は変更申請で対応する。

（上記の場合は、必ず説明文書に提供目的等を記載のこと）

□2）任意様式※を提供記録とし、「その他報告」か「実施状況報告」で対応する。

＊この際は報告する様式を添付してください

※厚労省 HP の様式を参考にすること。

http://www.mhlw.go.jp/stf/seisakunitsuite/bunya/hokabunya/kenkyujigyou/i-kenkyu/

□3）新規申請時は、本研究計画書を提供記録とするが、その後は 2）の対応とする。

＊この際は報告する様式を添付してください

□4）その他（具体的に：例：「提供に関する契約書（MTA（material transfer agreement）、

DTA（data transfer agreement）等）」を用いる。 ）

②提供記録の保管方法

・提供記録の保管場所：金沢大学保健管理センターの教員研究室の鍵のかかる保管庫のノートPC に電子データとして保存。個人を特定できない測定データに関しては、セキュリティを強化したファイルサーバ上に保存し、個人ごとにユーザー名、パスワードを管理するとともに、アクセスログを記録する。

③提供元の機関名称：京セラ株式会社 みなとみらいリサーチセンター

提供元の責任者名：瀧川知昭

④ 提供元のインフォームド・コンセントの方法：文章による説明とインフォームド・コンセントの取得

⑤ 提供元の研究対象者への情報公開：取得した情報の公開は行わない。

⑥提供を受ける試料・情報の項目：身長、体重、既往歴、服用薬などの研究対象者の基本データ（電子データ）、心理テスト調査票（紙データ）、心拍変動解析測定データ（電子データ）

⑦ 提供元の対応表の管理方法：

施設の個人情報管理者が適切に管理を行い外部への提供は行わない。

18．部局長への報告

■ 有害事象報告（随時）

■ 研究計画書からの重大な逸脱に関する報告（随時）

■ 実施状況報告（年1回）

■ 終了報告（研究終了時）

□ その他 （　　　　）

19．研究成果の帰属と結果の公表

本研究の結果として知的財産権が生じる可能性がある。その権利は国、研究機関、民間企業を含む共同研究機関及び研究従事者などに属し、研究対象者にはこの知的財産権は属さない。

20．研究実施体制

（1）研究代表者　吉川弘明（保健管理センター，教授）

（2）金沢大学における研究責任者及び研究分担者

研究責任者　　吉川弘明（保健管理センター，教授）
研究分担者　　足立由美（保健管理センター，教授）
　　　　　　　　馬場絢子（保健管理センター，助教）

（3）共同研究機関と研究責任者

研究機関：京セラ株式会社 みなとみらいリサーチセンター

研究責任者：瀧川知昭（ｺﾐｭﾆｹｰｼｮﾝｼｽﾃﾑ研究開発部・係責任者）
研究分担者：　山口 優哉 （ｺﾐｭﾆｹｰｼｮﾝｼｽﾃﾑ研究開発部・社員）
中井 若菜 （同上）
須藤 大貴 （同上）
稲田 明 （同上）
業務内容：データ収集、データ解析、ミリ波センサの改良・調整、研究対象者のリクルート，検査会場の提供

２１．文献

1. Adachi Y, Yoshikawa H, Yokoyama S, Iwasa K. Characteristics of university students supported by counseling services: Analysis of psychological tests and pulse rate variability. PLOS ONE. 2020;15(8):e0218357. doi: 10.1371/journal.pone.0218357.

2. Sullivan MB, Erb M, Schmalzl L, Moonaz S, Noggle Taylor J, Porges SW. Yoga Therapy and Polyvagal Theory: The Convergence of Traditional Wisdom and Contemporary Neuroscience for Self-Regulation and Resilience. Front Hum Neurosci. 2018;12:67. Epub 2018/03/15. doi: 10.3389/fnhum.2018.00067. PubMed PMID: 29535617; PubMed Central PMCID: PMCPMC5835127.

3. Heart rate variability. Standards of measurement, physiological interpretation, and clinical use. Task Force of the European Society of Cardiology and the North American Society of Pacing and Electrophysiology. Eur Heart J. 1996;17(3):354-81. Epub 1996/03/01. PubMed PMID: 8737210.

4. Hinde K, White G, Armstrong N. Wearable Devices Suitable for Monitoring Twenty Four Hour Heart Rate Variability in Military Populations. Sensors (Basel). 2021;21(4). Epub 2021/02/10. doi: 10.3390/s21041061. PubMed PMID: 33557190; PubMed Central PMCID: PMCPMC7913967.

5. Müller AM, Wang NX, Yao J, Tan CS, Low ICC, Lim N, et al. Heart Rate Measures From Wrist-Worn Activity Trackers in a Laboratory and Free-Living Setting: Validation Study. JMIR Mhealth Uhealth. 2019;7(10):e14120. Epub 2019/10/04. doi: 10.2196/14120. PubMed PMID: 31579026; PubMed Central PMCID: PMCPMC6777285.

２２．研究に関する業務の一部を委託する場合の、当該業務内容及び委託先の監督方法
■委託しない
□委託する

２３．モニタリングについて

■該当なし
□該当あり

24．監査について
□該当なし
■該当あり
京セラ株式会社による書類審査

25．研究事務局・相談窓口
〒920-1192　石川県金沢市角間町　金沢大学保健管理センター　足立由美
電話: 076-264-5254
FAX: 076-234-4044

…………………………………………………………

その他注意事項
