# Supplementary material for: Heart rate variability versus visual analog scale for objective and subjective mental fatigue detection: A randomized controlled trial
Source: PLOS Ment Health. 2025 Jan 24;2(1):e0000240. doi: 10.1371/journal.pmen.0000240 (PMC12798607; doi:10.1371/journal.pmen.0000240)
Supplement: S2 Text — (PDF) [file pmen.0000240.s004.pdf]

Research Protocol

1. Project title: Research on understanding mental and physical conditions through heart rate variability analysis

2. Overview, purpose, and significance of the research

Maintaining good physical and mental health while studying and working is critical in today's stressful society. In particular, the novel coronavirus pandemic has drastically changed the way people study and work, and maintaining and improving mental health related to this has become an issue. By the way, our research on university students revealed that students with mental health problems have the following characteristics. 1) working memory tends to be low, although within the normal range; 2) traits of autism spectrum disorder are high, especially in males; 3) low resilience; 4) personality traits are high in anxiety; 5) gender In both cases, it is difficult to feel the quality of life (QOL) in social life, 6) in men, indicators of QOL in terms of mental health are low, and 7) in terms of autonomic nervous function, sympathetic nervous tension is high even at rest [1]. Based on these results, the current research plan is to 1) clarify an index of heart rate variability effective for monitoring workers' mental health and 2) use a non-restrained heart rate recording device using a millimeter wave sensor (a joint research project). This study aims to examine whether the device (developed by Kyocera Corporation) has the same performance as conventional devices that analyze the heart's electrical activity. If this device is commercialized, it will be treated as a "research device." Furthermore, this research is not intended to evaluate the performance of medical devices based on the Pharmaceutical and Medical Devices Act.

3. Scientific rationality and basis of research

This study is a randomized controlled study using a non-invasive intervention. Use mental exertion load (calculation) as an intervention. The characteristics of students with mental health problems are as mentioned above [1], but among them, resilience is essential as an ability to perform work under stress. We also consider fatigue and vitality important indicators when performing work and evaluate these items using psychological tests.

The basis for using heart rate variability analysis in the evaluation of this study is based on the following report. According to the Polyvagal theory proposed by Porges, SW, the activity of the vagus nerve, composed of new myelinated nerves, enriches people's sense of security and social relationships, improving their resilience [2]. Based on these findings, we planned to conduct a heart rate variability analysis focusing on parasympathetic nerve activity.

Autonomic nerve function analysis is performed by analyzing the R-R interval of electrocardiogram waveforms, which has been studied for some time and has guidelines from academic societies [3]. However, the measurement locations and conditions have been limited since it is difficult to carry out daily activities while wearing an electrocardiograph. Therefore, wearing a millimeter wave sensor is necessary, and we are investigating whether it is possible to obtain measurement results similar to those of conventional electrocardiogram recording devices.

4. Research subjects and selection policy

(1) Eligibility criteria

1) People who received annual health check-ups had no significant health problems.

2) Those who can provide written consent to participate in this study.

3) Persons who are 20 years of age or older at the time of obtaining consent

4) Include an equal ratio of men and women (ask the staffing company that dispatches the research subjects to ensure that the number of men and women is equal).

(2) Exclusion criteria

1) Persons with cardiac pacemakers

2) People with arrhythmia as a disease

3) Those taking drugs that affect autonomic nervous function (β blockers)

5. Target number and research implementation period

(1) A target number of 140 cases was randomly assigned to the intervention (continuous addition task) group and control (reading) group.

(2) Research period

Research period:	Approval date to March 3, 2025
Registration period:	Approval date to September 30, 2022
Observation period (follow-up period):	1 year after registration ends
Analysis period:	Approval date to March 3, 2025

*The ethics review committee will review collaborative research institutions under the regulations of each institution, and permission will be obtained from the head of the research institution.

The research start date for joint research institutions shall be when research implementation permission is granted for each institution.

6. research method

(1) Research design

Single-center prospective randomized controlled study

(2) Research method

The research headquarters will be located at the Kanazawa University Health Service Center (referred to as the Kanazawa Center from now on) to manage and analyze data. Subjects will be recruited by a staffing company at Kyocera Corporation Minato Mirai Research Center (Yokohama City, from now on referred to as Yokohama Center). Data will be collected in a room at a constant temperature in the Yokohama Center building or in a rental office. The Kanazawa Center Research Director and Co-Investigators will guide research execution via video conference as necessary. At the Yokohama Center, subjects' personal information and acquired data will be managed, and the management ledger (electronic data) will be stored in a locked storage cabinet on a laptop or storage medium. The principal investigator or co-investigator from Kyocera, a joint research institution, will provide written explanations and obtain consent. If there are any questions, the principal investigator or co-investigator from the Kanazawa Center will answer them via video conference. The selection of 1. continuous addition task and 2. control task (reading) during the intervention (85 min) will be done by drawing lots. The reading for the comparison task will be a light novel (e.g., Kenji Miyazawa, a collection of children's stories), which will be selected by the Kanazawa Center and mailed to the Yokohama Center. Use the same books during the research process.

The Kanazawa Center will analyze and evaluate psychological tests and heart rate variability data. The analysis results will be shared with the Yokohama Center using a file server. The file server sets each user's name and password and records access logs. Please note that no personally identifiable information is stored on the file server.

The following two types of measuring instruments are used for heart rate variability analysis and are recorded simultaneously.

1) Polar 's POLAR H10 heart rate sensor (Document 1) and POLAR VANTAGE V2 sports watch (Document 2).

This wearable device is a simple electrocardiograph using a chest sensor that records the electrocardiogram's R R interval. The data has been considered to have the same accuracy as an electrocardiograph [4, 5].

2) Kyocera Corporation Millimeter wave sensor

This non-contact heart rate recorder uses millimeter-wave radar technology (Document 3). (Please note that this device is under development and contains highly confidential technology, so specifications cannot be provided.)

See Document 4 for the measurement plan for research subjects.
Research methods may be added as the research progresses.
Psychological test results will not be disclosed to research subjects. The reasons for this are: 1) the results of psychological tests cannot be adequately communicated unless they are explained directly by a psychologist, and 2) some concerns that communicating the results of psychological tests for a limited purpose may lead to incorrect self-understanding.

7. Observation/inspection/report items
1) Information regarding the characteristics of the research subjects
Age, gender, height, weight, medical history (presence or absence of chronic headaches, etc.), diseases being treated, medications taken
2) Psychological test
See research observation and inspection schedule (Document 4)
3) Heart rate variability
Obtained from chest-worn sensor (POLAR H10) and millimeter wave sensor.

During measurements, the Yokohama Center's research director or co-researcher will be present and manage the process to ensure that the research progresses according to plan. Before implementation, sufficient meetings will be held at the Kanazawa Center and Yokohama Center to ensure that there are no procedure mistakes. A correspondence table will be created for the completed test forms, anonymized, and mailed to the Kanazawa Center. Co-researchers Adachi and Baba will analyze and score the questionnaire. Principal investigator Yoshikawa will analyze heart rate variability data using the Kubios analysis software and an algorithm developed by Kyocera Corporation. The measurement data will be shared with the Kyocera Center via a file server. Furthermore, for the Kanazawa Center's research director and collaborators to confirm that the research is being conducted appropriately, the Yokohama Center will record videos during research and share them with the Kanazawa Center.

8. Evaluation and reporting of adverse events
(1) Definition and reporting method of adverse events
□Not applicable
■Applicable

This prospective study involves a non-invasive, mild intervention (continuous addition work), but if any adverse events occur due to this intervention, necessary measures will be taken and reported to the department head.

(2) The burden on research subjects, the expected risks and benefits, a comprehensive evaluation, and measures to minimize the burden and risk.

① Expected profit

Participating in this study has no direct benefits to individual research subjects. The research results could be useful in developing tools to monitor workers' mental and physical health.

② Expected risks and disadvantages

This study is a randomized controlled trial with a non-invasive, mild intervention (calculation work), and we believe the risks are low. The disadvantage is that each measurement takes approximately 200 minutes, which means valuable time is lost. During the experiment, eating and drinking other than water is prohibited. Going to the toilet is possible between measurements and interventions if necessary. Because the research is conducted indoors in an environment with constant room temperature and brightness and no noise or stress, it is thought that there are few health risks from participating. If transportation costs to the research location are incurred, the research subjects will be responsible for their expenses.

If a research subject develops a health problem during the research, the test will be interrupted, and they will be placed in a separate room to rest. If the condition does not improve, a nearby medical institution will be used, and if any costs are incurred, Kyocera Corporation will cover them.

9. Evaluation item

(1) Main evaluation items:

Rate Variability (HRV) analysis results

(2) Secondary endpoints:

POMS2 score

Fatigue evaluation using the VAS method (Document 5)

S -H Resilience Test Score

W HO QOL26 score

Definition of test statistics (see attached measurement plan supplement)

･Psychological test scores: Psychological test-1, psychological test-2 scores, and the difference between them.

- Heart rate variability analysis indexes: Heart rate variability data will be measured continuously from the start (observation period (resting sitting position, 5 min)) until the end of the test. The test statistics are the power spectrum analysis values in each section of Record 1 and Record 2 and the difference.

10. statistical matters

The objective number of participants was determined using experimental design statistics from our past research results [1]. This study performed an unpaired comparison between two groups. After checking whether the data had a normal distribution, we checked whether the data had homoscedasticity. In the case of normal distribution and homoscedasticity, Student's t-test was performed, and in the case of non-uniform variance, Welch's t-test was performed. For non-normally distributed data, a Wilcoxon-Mann-Whitney test was performed.

The analysis items were 1) changes in the heart rate variability analysis index and psychological test scores before and after the intervention and 2) evaluation of the phasing between the heart rate variability analysis index Polar H10 and the millimeter wave sensor. The statistical method in 1) involves a comparison between two paired groups, and after confirming the normality of the data, an appropriate statistical analysis method compatible with parametric or non-parametric methods is selected. Regarding 2), a correlation analysis will be performed.

11. Completing and reporting the case report form

There are no defaults. Create a checklist for the research process and time management.

12. Ethical considerations

(1) Ethical guidelines and laws to comply with

All those involved in this research must comply with the "World Medical Association Declaration of Helsinki" and the "Ethical Guidelines for Medical Research Involving Human Subjects" (Ministry of Education, Culture, Sports, Science and Technology/Ministry of Health, Labor and Welfare), which all medical research involving human subjects must comply.) Please read and understand the contents carefully before conducting research.

(2) How to protect personal information

Those involved in research will comply with applicable laws and ordinances regarding protecting the personal information of research subjects. In addition, those involved must make every effort to protect research subjects' personal information and privacy and not divulge personal information obtained while conducting this research without justifiable reason. The same shall apply even after the person concerned retires from his or her position.

When handling samples related to research implementation, sufficient consideration will be given to protecting the confidentiality of test subjects. When sending psychological tests (paper or booklet) from the Yokohama Center to the Kanazawa Center, a number will be used, and sufficient care will be taken to ensure that the test subject's personal information is not leaked to outside parties. Personal information and anonymized correspondence tables are stored as electronic files on separate notebook PCs, and the notebook PCs are kept in a locked desk at the

Yokohama Center, with strict precautions to prevent leakage, theft, or loss. Measurement data that does not include personal information is stored on a file server, a password is set for each user who accesses it, and access logs are managed. Ayako Baba (Kanazawa University Health Management Center) will be appointed personal information administrator.
When publishing research results at academic conferences, etc., prevent individuals from being identified and protect anonymity.
After the research is discontinued or completed, electronic data and experiment/observation notes related to the research will be stored for ten years, and other research data will be stored for five years, starting from the latest conference presentations and paper presentations.

13. Procedures for obtaining informed consent

Kyocera Corporation's research director or co-researcher will provide the research subject with a consent explanation document that the ethics review committee has approved in advance, provide a sufficient written explanation, and decide whether or not to participate in the research. , Obtain written consent from research subjects to participate in the research freely. If the explanation is insufficient, our university's research director or co-investigator will supplement the explanation via video conference.

14. Regarding the cost burden incurred by research subjects

It is thought that there will be no financial burden on the research subjects. Pay compensation according to regulations.

15. Regarding funding and conflicts of interest related to this research

This research is a joint research with Kyocera Corporation, and the company will bear the cost. The equipment used in the research (millimeter wave sensor manufactured by Kyocera Corporation) will be provided by Kyocera Corporation. When conducting and publishing this research, conflicts of interest will be appropriately managed, neutrality and transparency will be maintained, and the research will be maintained appropriately. In addition, the person in charge of this study will submit the necessary information to the Kanazawa University Clinical Research Conflict of Interest Management Committee following the "Kanazawa University Clinical Research Conflict of Interest Management Policy" and obtain its review and approval. When publishing research results, the guidelines of the academic society and journal in which the results will be published shall be complied with, and accurate circumstances shall be disclosed through self-reporting.

16. Regarding changes to the implementation plan

If, as the research progresses, it becomes necessary to change the plan in terms of research

content, research organization, period, etc., such changes will be made with the approval of the Medical Ethics Review Committee.

17. About samples and information

(1) Types of specimens and information, storage, recording, and disposal

A. Samples obtained from the human body

■Not applicable

□Applicable

Sample type:

Regarding storage/disposal:

Regarding secondary use of samples and information:

About the person responsible for preservation:

B. information

□Not applicable

■Applicable

Types of information: Basic data of research subjects such as age, gender, height, weight, medical history, medications taken (electronic data), psychological test questionnaire (paper data, electronic data), heart rate variability data (electronic data))

At the Yokohama Center, we will collect name and phone numbers.

Regarding storage/disposal:

The research director will instruct the co-researchers to store the information properly following the prescribed storage method and will perform necessary management to prevent information leakage, theft, loss, etc. Electronic data and experiment/observation notes will be stored for ten years from the end of the research or after the publication of papers, etc., and other research data will be stored for five years before being destroyed. The joint research institution (Yokohama Center) that provides the information will store and destroy it appropriately based on its rules.

Regarding the secondary use of samples and information:

Samples and information of research subjects obtained in this study may be used for future research that will not be specified at the time of consent. In that case, the new research plan will be reviewed by the University's Ethics Review Committee, and the research will be explained separately to the research subjects before implementation.

About the person responsible for preservation: Hiroaki Yoshikawa, the research director, will preserve information.

(2) Records of exchange of specimens and information with other institutions
[When providing samples and information to other organizations (including provision through partial outsourcing of work)]
■Not applicable
□Applicable
① How to create provision records
*Please be sure to check with the provider before providing the information.
□1) This research plan will be used as a record of provision, and any changes will be handled by applying for changes.
(In the above case, be sure to state the purpose of the provision etc., in the explanatory document.)
□2) Use the optional format* as a record of provision, and respond with "other reports" or "implementation status reports."
*In this case, please attach the reporting form.
*Please refer to the format on the Ministry of Health, Labor and Welfare website.
http://www.mhlw.go.jp/stf/seisakunitsuite/bunya/hokabunya/kenkyujigyou/i-kenkyu/
□3) When making a new application, this research plan will be used as the provision record, but after that, 2) will be followed.
*In this case, please attach the reporting form.
□4) Others (specifically: e.g., use a "provision agreement (MTA (material transfer agreement), DTA (data transfer agreement), etc.)"
② How to store provision records
・Storage location of provision records:
Name of the institution receiving the offer:
Name of person responsible for provision:
Items of samples and information to be provided:

[When receiving specimens and information from other institutions]
□Not applicable

■Applicable
① How to create provision records
*Please be sure to check with the provider regarding availability.
■1) This research plan will be used as a record of provision, and any changes will be handled by applying for changes.
(In the above case, be sure to state the purpose of the provision etc., in the explanatory document.)
□2) Use the optional format* as a record of provision, and respond with "other reports" or "implementation status reports."
*In this case, please attach the reporting form.
*Please refer to the format on the Ministry of Health, Labor and Welfare website.
http://www.mhlw.go.jp/stf/seisakunitsuite/bunya/hokabunya/kenkyujigyou/i-kenkyu/
□3) When making a new application, this research plan will be used as the provision record, but after that, 2) will be followed.
*In this case, please attach the reporting form.
□4) Others (specifically: e.g., use a “provision agreement (MTA (material transfer agreement), DTA (data transfer agreement), etc.”)
② How to store provision records
Saved as electronic data on a laptop in a locked storage room in the faculty laboratory of the Kanazawa University Health Management Center. Measurement data that cannot identify individuals will be stored on a file server with enhanced security, user names and passwords will be managed for each individual, and access logs will be recorded.
③ Name of provider institution: Kyocera Corporation Minato Mirai Research Center
Provider name: Tomoaki Takigawa
④ Method of obtaining informed consent from provider: Written explanation and obtaining informed consent
⑤ Disclosure of information to the research subjects of the provider: The obtained information will not be disclosed.
⑥ Items of samples and information to be provided: Basic data of research subjects such as height, weight, medical history, and medications (electronic data), psychological test questionnaire (paper data), heart rate variability data (electronic data)
⑦ How to manage provider correspondence table:
The facility's personal information manager will manage the information appropriately and not provide it to outside parties.

18. Report to department head
■ Adverse event reporting (as needed)

■ Reports on significant deviations from the research plan (as needed)

■ Implementation status report (once a year)

■ Completion report (at the end of the research)

□ Others ( )

19. Attribution of research results and publication of results

Intellectual property rights may arise as a result of this research. Those rights belong to the government, research institutions, joint research institutions, private companies, and research personnel; these intellectual property rights do not belong to the research subjects.

20. Research implementation system

(1) Research representative Hiroaki Yoshikawa (Health Management Center, Professor)

(2) Research director and research collaborator at Kanazawa University

Research Director Hiroaki Yoshikawa (Health Management Center, Professor)

Co-researcher Yumi Adachi (Health Management Center, Professor)

Ayako Baba (Health Management Center, Assistant Professor)

(3) Joint research institution and research director

Research institution: Kyocera Corporation Minato Mirai Research Center

Research Director: Tomoaki Takigawa (Communication System Research and Development Department/Section Manager)

Researcher: Yuya Yamaguchi (Communication System Research and Development Department/Employee)

Wakana Nakai (same as above)

Daiki Sudo (same as above)

Akira Inada (same as above)

Business details: Data collection, data analysis, improvement and adjustment of millimeter wave sensors, recruitment of research subjects, provision of testing venues

21. literature

1. Adachi Y, Yoshikawa H, Yokoyama S, Iwasa K. Characteristics of university students supported by counseling services: Analysis of psychological tests and pulse rate variability. PLOS ONE. 2020;15(8):e0218357. doi: 10.1371/journal.pone.0218357.

2. Sullivan MB, Erb M, Schmalzl L, Moonaz S, Noggle Taylor J, Porges SW. Yoga Therapy and Polyvagal Theory: The Convergence of Traditional Wisdom and Contemporary

Neuroscience for Self-Regulation and Resilience. Front Hum Neurosci. 2018;12:67. Epub 2018/03/15. doi: 10.3389/fnhum.2018.00067. PubMed PMID: 29535617; PubMed Central PMCID: PMCPMC5835127.

3. Heart rate variability. Standards of measurement, physiological interpretation, and clinical use. Task Force of the European Society of Cardiology and the North American Society of Pacing and Electrophysiology. Eur Heart J. 1996;17(3):354-81. Epub 1996/03/01. PubMed PMID: 8737210.

4. Hinde K, White G, Armstrong N. Wearable Devices Suitable for Monitoring Twenty Four Hour Heart Rate Variability in Military Populations. Sensors (Basel). 2021;21(4). Epub 2021/02/10. doi: 10.3390/s21041061 . PubMed PMID: 33557190; PubMed Central PMCID: PMCPMC7913967.

5. Müller AM, Wang NX, Yao J, Tan CS, Low ICC, Lim N, et al. Heart Rate Measures From Wrist-Worn Activity Trackers in a Laboratory and Free-Living Setting: Validation Study. JMIR Mhealth Uhealth. 2019; 7(10):e14120. Epub 2019/10/04. doi: 10.2196/14120. PubMed PMID: 31579026; PubMed Central PMCID: PMCPMC6777285.

22. When part of research-related work is outsourced, the content of the work and the method of supervising the outsourcing company

■Do not outsource

□Design

23. About monitoring

■ Not applicable

□Applicable

24. About audit

□Not applicable

■Applicable

Document examination by Kyocera Corporation

25. Research office/consultation desk

Yumi Adachi, Kanazawa University Health Management Center, Kakuma-cho, Kanazawa City, Ishikawa Prefecture 9 20-1192

Phone: 076-264-5254

FAX: 076-234-4044

……………………………………………………………

Other notes
